# Global and Geographically and Temporally Weighted Regression Models for Modeling PM_2.5_ in Heilongjiang, China from 2015 to 2018

**DOI:** 10.3390/ijerph16245107

**Published:** 2019-12-14

**Authors:** Qingbin Wei, Lianjun Zhang, Wenbiao Duan, Zhen Zhen

**Affiliations:** 1 School of Forestry, Northeast Forestry University, Harbin 150040, China; wqb.0816@aliyun.com (Q.W.); dwbiao88@126.com (W.D.); 2 Department of Forest and Natural Resources Management, State University of New York College of Environmental Science and Forestry, One Forestry Drive, Syracuse, New York, NY 13210, USA; lizhang@esf.edu; 3 Key Laboratory of Sustainable Forest Ecosystem Management-Ministry of Education, Northeast Forestry University, Harbin 150040, China

**Keywords:** GTWR, GWR, TWR, LMM, PM_2.5_, air pollutants

## Abstract

*Objective:* This study investigated the relationships between PM_2.5_ and 5 criteria air pollutants (SO_2_, NO_2_, PM_10_, CO, and O_3_) in Heilongjiang, China, from 2015 to 2018 using global and geographically and temporally weighted regression models. *Methods:* Ordinary least squares regression (OLS), linear mixed models (LMM), geographically weighted regression (GWR), temporally weighted regression (TWR), and geographically and temporally weighted regression (GTWR) were applied to model the relationships between PM_2.5_ and 5 air pollutants. *Results:* The LMM and all GWR-based models (i.e., GWR, TWR, and GTWR) showed great advantages over OLS in terms of higher model R^2^ and more desirable model residuals, especially TWR and GTWR. The GWR, LMM, TWR, and GTWR improved the model explanation power by 3%, 5%, 12%, and 12%, respectively, from the R^2^ (0.85) of OLS. TWR yielded slightly better model performance than GTWR and reduced the root mean squared errors (RMSE) and mean absolute error (MAE) of the model residuals by 67% compared with OLS; while GWR only reduced RMSE and MAE by 15% against OLS. LMM performed slightly better than GWR by accounting for both temporal autocorrelation between observations over time and spatial heterogeneity across the 13 cities under study, which provided an alternative for modeling PM_2.5_. *Conclusions:* The traditional OLS and GWR are inadequate for describing the non-stationarity of PM_2.5_. The temporal dependence was more important and significant than spatial heterogeneity in our data. Our study provided evidence of spatial–temporal heterogeneity and possible solutions for modeling the relationships between PM_2.5_ and 5 criteria air pollutants for Heilongjiang province, China.

## 1. Introduction

Air pollutants can be emitted from anthropogenic and natural sources and may be either emitted directly (primary pollutants) or formed in the atmosphere (as secondary pollutants) [1]. They may be transported or formed over long distances and have influences on human health, ecosystems, the built environment, and climate in large areas. It has been confirmed by extensive epidemiological studies that air pollution is closely associated with increased risks of mortality or morbidity for cardiovascular and respiratory diseases [2,3,4,5]. It was reported that air pollution ranked the 7^th^ killer to the global public health and contributed to 3.2 million and 4.2 million premature deaths Worldwide in 2010 and 2016, respectively [6,7]. The European Environment Agency estimates that about 30% of Europe’s urban population is still exposed to air pollution concentrations exceeding the European Union air quality standards [1,8]. With the rapid urbanization and industrialization, China has experienced a serious challenge of preventing and controlling air pollution as well. About 1.2 million premature deaths in 2010 and 25 million disability-adjusted life-years annually in China resulted from ambient or outdoor air pollutions [9]. Ambient particulate matter pollution has become the 4th killer to the health of Chinese people in 2010, after dietary risk, high blood pressure, and smoking [6]. It is of great importance to prevent and control air pollution for constructing ecological civilization in China.

In 2012, the Chinese Ministry of Environmental Protection (MEP) released the revised Ambient Air Quality Standards and defined the 6 criteria air pollutants including the particular matter with aerodynamic diameter of 10 μm or less (PM_10_), the particular matter with aerodynamic diameter of 2.5 μm or less (PM_2.5_), ozone (O_3_), nitrogen dioxide (NO_2_), and sulfur dioxide (SO_2_), and carbon monoxide (CO) [10]. To mitigate the conflicts between air pollution and public health, the Chinese State Council issued the Air Pollution Prevention and Control Action Plan (APPCAP) in September 2013 and proposed a goal of reducing PM_2.5_ by 10% in major cities by the end of 2017 compared to 2012 levels [11]. The Plan was a milestone of air pollution prevention and control efforts in China and considered the most stringent air pollution control policy in China to date [12]. Although the improvements to air quality in most cities in recent years, decreasing PM_2.5_ in the Northern Plain of China is still a tough challenge and requires strong transregional collaboration [13]. Most studies focused on the levels of air pollution in the Beijing-Tianjin-Hebei region, the Yangtze River Delta, and the Pearl River Delta, which are the most developed regions in China in terms of a high level of per capita GDP and high population density (e.g., [14,15,16,17,18]). There is no adequate attention paid to the air pollution problems in northern China compared to above-developed regions, especially in the Heilongjiang Province.

To clarify the pollution level, characteristics, and main sources of particulate matter, the Heilongjiang government began to investigate the sources of atmospheric particulate matter within the province in 2013 [19]. It found that the main pollutants in the cities of Heilongjiang Province were inhalable particulate matter (PM_10_) and fine particulate matter (PM_2.5_). In particular, the increase in coal-fired emissions during the heating season (late October, November, December, January, February, and March) significantly increased the concentration of PM_2.5_. The local sources of PM_10_ and PM_2.5_ in Heilongjiang Province are mainly from coal-fired, followed by motor vehicle exhaust and dust. The contributions of different sources have certain seasonal variations [19]. Although the sources of air pollutants were explored, the spatial and temporal variation characteristics of different air pollutants in Heilongjiang Province were barely investigated.

Space and time are significant determinants of PM_2.5_. Similar to Tobler’s (1970) first law of geography that is “everything is related to everything else, but near things are more related than distant things” [20], many geographic processes (such as air pollution) followed similar rules in the temporal domain. Over the last two decades, researchers developed various statistical models to deal with spatial and/or temporal heterogeneities. Geographically weighted regression (GWR) was designed to extend the traditional global model fitted by ordinary least squares (OLS) and could effectively deal with spatial heterogeneity and autocorrelation in model errors [21,22,23]. GWR belongs to local modeling techniques and fits a regression model at each geographic location based on the neighbors within a specific bandwidth and distance-dependent weight function. In recent years, researchers have extended GWR to a temporal dimension for spatiotemporal modeling, which is called geographically and temporally weighted regression (GTWR) [24,25]. GTWR deals with both spatial and temporal nonstationarities simultaneously by constructing a weight matrix based on spatiotemporal distance. GTWR greatly expands the boundary of local modeling techniques, and has been applied in various fields, such as environment, economics, sociology, and so on [24,26,27,28,29,30]. As a special case of GTWR, GWR ignores the temporal non-stationarity, while TWR (temporally weighted regression) ignores the spatial non-stationarity thus that GTWR integrates GWR and TWR into one uniform framework.

Besides GTWR, linear mixed models (LMM) can be applied to deal with both temporal autocorrelations and spatial heterogeneity problems. LMM can account for the sources of heterogeneity and dependence in the data by using the random-effects and model temporal autocorrelations by using appropriate covariance matrices for the model residuals, thereby aiding in statistical inference [31]. LMM can improve model fitting and performance if a random variation is focused, particularly in the studies of ecological heterogeneity or the heritability of discrete characters [32]. However, LMM is not widely applied for air pollution data but particularly suitable to this study because it can deal with air pollutants as the fixed-effects and cities (spatial) as the random-effects within longitudinal observations (temporal). Therefore, LMM is an appropriate alternative to model PM_2.5_ in this study.

Given the need for a better understanding of spatial–temporal heterogeneity between the criteria air pollutants in Heilongjiang Province, spatial–temporal statistical techniques present an important tool to quantitatively describe the amount of air pollution at particular locations at specific times. The objective of this study was to model PM_2.5_ with the 5 criteria air pollutants (SO_2_, NO_2_, PM_10_, CO, and O_3_) in Heilongjiang Province, China, from 2015 to 2018 using global and geographically and temporally weighted regression models. Specifically, this study implemented 5 models, including ordinary least square regression (OLS) that was applied as the benchmark for model comparisons, linear mixed model (LMM) that considered different cities (or regions) as the random-effects and repeated measurements over time, traditional geographically weighted regression (GWR), temporally weighted regression (TWR), and geographically and temporally weighted regression (GTWR). The goodness-of-fit, prediction accuracy, uncertainty accuracy of models, and model residuals were evaluated and compared based on corrected Akaike’s information criterion (AICc), adjusted coefficient of determination ($R_{a}^{2}$), and root mean squared errors (RMSE), mean absolute error (MAE), normal (Z) scores, and Moran’s I of the model residuals. Understanding the spatial and temporal heterogeneity of various air pollutants can assist in the PM_2.5_ prediction and air pollutants control management in the future.

## 2. Materials and Methods

### 2.1. Study Area and Data

**Figure 1:**
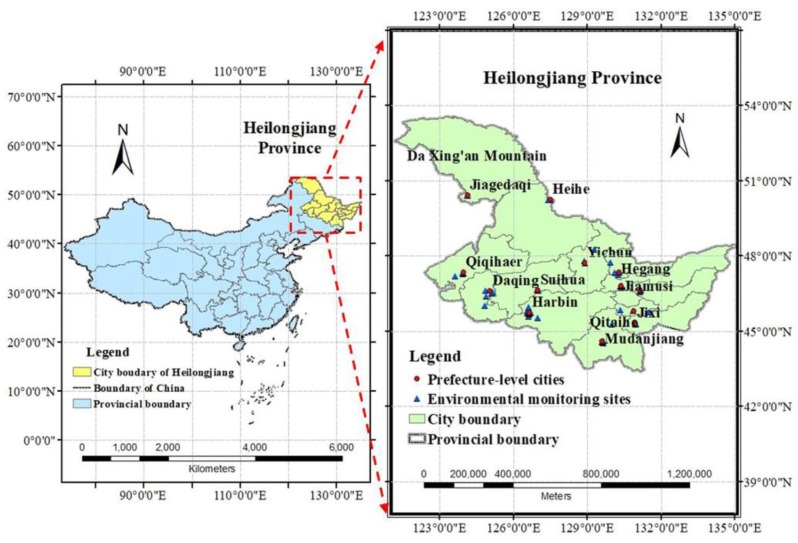
The geographical location of the study area: Heilongjiang Province, P.R. China (including the Da Xing’an Mountain). Note: Jiagedaqi is a special residential area where the environmental monitoring site of Da Xing’an Mountain region is located in.

Heilongjiang Province is located in the Northeast of China between 43°26′ N and 53°33′ N Latitude, and 121°11′ E and 135°05′ E Longitude. It has 13 prefecture-level administrative regions, including 12 prefecture-level cities (i.e., Harbin, Qiqiha’er, Mudanjiang, Jiamusi, Daqing, Jixi, Shuangyashan, Yichun, Qitaihe, Hegang, Suihua, and Heihe) and 1 region (Da Xing’an Mountain) with a total area of 473,000 km^2^ (Figure 1). Generally, the terrain of Heilongjiang is high in the northwest, north, and southeast, low in the northeast and southwest, and is mainly composed of mountains, terraces, plains, and water. The elevations of mountains, terraces and plains are between 300 and 1000 m (accounting for 58% of the total area), between 200 and 350 m (14%), and between 50 and 200 m (28%), respectively. Heilongjiang Province belongs to the continental monsoon climate of cold temperate and temperate zone with a short frost-free period and has large regional differences in climate. The main characteristics are low-temperature and drought in spring, warm and rainy in summer, dry and early-frost in autumn, and long cold in winter. The precipitation is abundantly affected by the southeast monsoon in summer and insufficient controlled by the dry and cold northwest wind in winter, which presents obvious monsoon characteristics.

In this study, 6 criteria air pollutants, including SO_2_, NO_2_, PM_10_, CO, O_3_, and PM_2.5,_ were obtained from the Environmental Monitoring Station of Heilongjiang Province and had already aggregated into the 12 prefecture-level cities and 1 region based on the 56 environmental monitoring sites in Heilongjiang Province. All the pollutants were recorded by μg/m^3^, except CO (mg/m^3^). The daily records of the criteria air pollutants were aggregated into weekly measurements of the 13 cities (region) of Heilongjiang Province from 2015 to 2018 (i.e., 210 weeks per city or region, and 2730 records in total). The descriptive statistics of the 6 air pollutants are summarized in Table 1.

**Figure 2:**
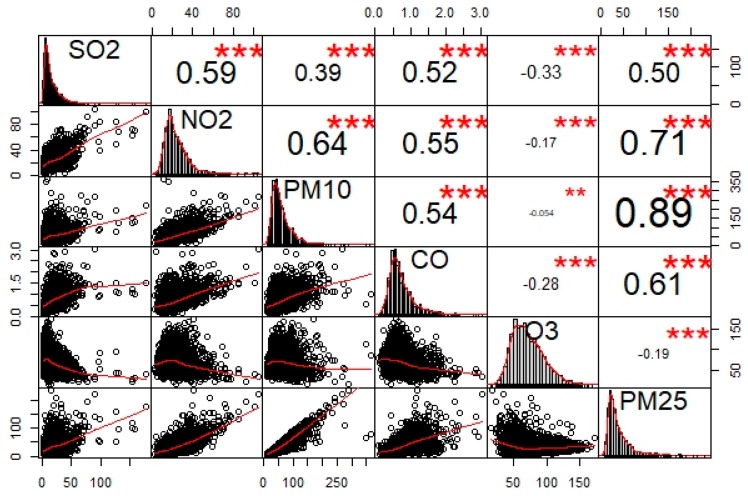
Pearson correlation coefficients (ρ) between the 6 air pollutants under study. The diagonal plots are the frequency distributions of SO_2_, NO_2_, PM_10_, CO, O_3_, and PM_2.5_.

**Figure 3:**
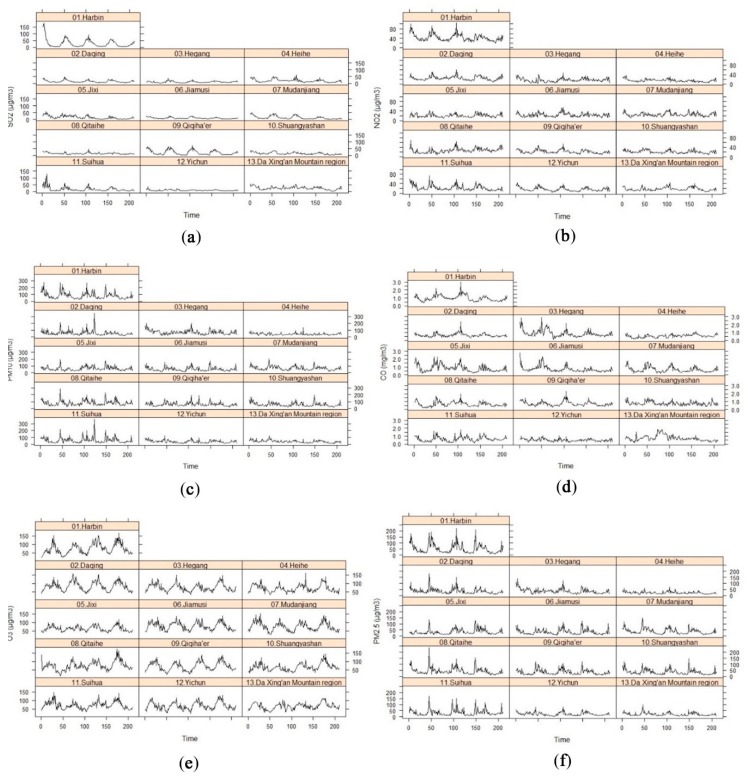
Variability of the 6 criteria air pollutants in the 13 cities (region) of Heilongjiang Province from 2015 to 2018 (210 weeks in total) (**a**) SO_2_, (**b**) NO_2_, (**c**) PM_10_, (**d**) CO, (**e**) O_3_, and (**f**) PM_2.5_.

Figure 2 presents the matrix of Pearson correlation coefficients (ρ) of the 6 air pollutants. It showed that PM_2.5_ was highly correlated (ρ >0.5) with PM_10_, NO_2_, CO, and SO_2_; whereas O_3_ was weakly (−0.25 < ρ < 0) to moderately (−0.5 < ρ < −0.25) correlated with the group of PM_10_, NO_2_ and PM_2.5_, and the group of SO_2_ and CO, respectively. Figure 3 presents the variability of the 6 criteria air pollutants cross locations (12 prefecture-level cities and 1 region) and periods (210 weeks from 2015 to 2018). There were obvious seasonal trends for all the air pollutants in most cities (region), especially in Harbin, Qiqiha’er, and Suihua. On the other hand, Heihe, Yichun, and Da Xing’an Mountain region showed relatively stable trends for all pollutants, except O_3_ compared to other cities. It indicated both spatial and temporal variability visually existing in Heilongjiang Province.

### 2.2. Methods

#### 2.2.1. OLS and LMM

Traditional ordinary least square (OLS) regression was used as the benchmark for model comparisons as follows:

(1) $$Y_{i}=\beta_{0}+\beta_{1}X_{1}+\beta_{2}X_{2}+\beta_{3}X_{3}+\beta_{4}X_{4}+\beta_{5}X_{5}+\varepsilon_{i}$$

where *Y_i_* is the response variable (i.e., PM_2.5_ in this study), where *i* = 1, 2, …, *n*; *β*_0_ ~ *β*_5_ are the regression coefficients to be estimated from the data; the predictors *X*_1_ ~ *X*_5_ represent SO_2_, NO_2_, PM_10_, CO, and O_3_, respectively; and ε*_i_* is the random error term following the normal distribution with zero mean and constant variance, i.e., N(0, σ^2^I), with I denoting an *n* × *n* identity matrix.

The model coefficient vector *β^T^* = [*β*_0_, *β*_1_, …, *β*_5_] of OLS is estimated by

(2) $$\hat{\beta }={\left(X^{T}X\right)}^{-1}X^{T}Y$$

where the superscript *T* denotes the transpose of a matrix, *X* and *Y* are the vectors of predictors and response variables, respectively. The OLS regression represents a universal or constant relationship between predictors and response variables across the entire study area.

The traditional OLS model was not only a global model but also a fixed-effects model for the predictors (SO_2_, NO_2_, PM_10_, CO, and O_3_), which were the only levels or factors under consideration for the statistical inference. In contrast, the random-effects were the factors that were randomly selected from an infinite population of the possible levels or factors and could vary if the experiment was implemented for another time thus that the statistical inference was direct towards the entire population of factor levels, not just those “random” levels that were incorporated into the experiment. In this study, the 13 cities (region) were considered as the random-effects because they were one subset of the cities in Heilongjiang Province. Thus, the linear mixed model was used to incorporate both fixed-effects (SO_2_, NO_2_, PM_10_, CO, and O_3_) and random-effects (13 cities), as well as account for time series observations across the 4 year periods. LMM was an extension of the linear models, and can be expressed as:

(3) Y=Xβ+Zγ+ε

where *Y* is an *n* × 1 column vector of the response variable, *X* is an *n* × *p* matrix of the (*p* − 1) predictors with the first column of 1 for estimating the intercept coefficient, *n* is the number of sample observations, *p* is the number of fixed-effects parameters, *β* is an *p* × 1 vector of unknown fixed-effects parameters, *Z* is a known *n* × *q* design matrix for the *q* random-effects, *γ* is an *q* × 1 vector of unknown random-effects parameters, and *ε* is an *n* × 1 vector of the random model errors. LMM follows several assumptions:

(4) $$\{\begin{array}{c} E\left(\gamma \right)=0\;\mathrm{and}\;\mathrm{Var}\left(\gamma \right)=G\;\\ E\left(\varepsilon \right)=0\;\mathrm{and}\;\mathrm{Var}\left(\varepsilon \right)=R\\ \mathrm{Cov}\left(\gamma ,\varepsilon \right)=0,\;\;\;\gamma ,\;\varepsilon \sim Normal\\ \mathrm{Var}\left(Y\right)=V=ZGZ^{\prime }+R \end{array}$$

where E(∙), Var(∙), and Cov(∙) denote expectation, variance, and covariance, respectively; “*Normal*” represents “following a normal distribution”. The variance of *Y* can be estimated by random-effects design matrix Z, and covariance matrices G and R. The estimates of the fixed-effects and random-effects parameters can be expressed by Equations (5) and (6), respectively,

(5) $$\hat{\beta }={\left(X^{\prime }{\hat{V}}^{-1}X\right)}^{-1}X^{\prime }{\hat{V}}^{-1}Y$$

(6) $$\hat{\gamma }=\hat{G}Z^{\prime }{\hat{V}}^{-1}\left(Y-X\hat{\beta }\right)$$

where $\hat{G}$, $\hat{R}$, and $\hat{V}$ are the reasonable estimates of G, R, and V, respectively, from the data.

#### 2.2.2. GWR Model and Parameter Estimation

GWR model extends the traditional ordinary least square regression from a global to local framework [24], and can be expressed as follows:

(7) $$Y_{i}=\beta_{0}\left(u_{i},v_{i}\right)+\sum_{k=1}^{p-1}\beta_{k}\left(u_{i},v_{i}\right)X_{ik}+\varepsilon_{i}\;\;\;\;\;\;\;i=1,\;2,\ldots ,n$$

where *Y_i_* is the response variable, (*u_i_*, *v_i_*) denotes the coordinates of the location *i* in space, *β*_0_(*u_i_*,*v_i_*) and *β_k_*(*u_i_*,*v_i_*) represent the intercept and a set of (*p* − 1) slope parameters at the location *i*, respectively. *X_ik_* represents a set of (*p* − 1) predictors (*k* = 1, 2, …, *p*−1) at the *i*th location, *p* is the total number of parameters to be estimated, *ε_i_* is the error term of location *i*. Comparing to the “fixed” coefficient estimates of the global OLS model (Equation (1)), GWR captures the spatial heterogeneity using varied parameter estimates over space.

GWR follows Tobler’s first law of geography [20], and is calibrated using a locally weighted least squares approach, and the estimation of parameters is obtained by the following equation:

(8) $$\hat{\beta }\left(u_{i},v_{i}\right)={\left(X^{T}W\left(u_{i},v_{i}\right)X\right)}^{-1}X^{T}W\left(u_{i},v_{i}\right)Y$$

where *W*(*u_i_*,*v_i_*) is an *n × n* weight matrix whose off-diagonal elements are zero and diagonal elements denote the geographical weighting of the neighboring observations for the focal observation *i* as follows:

(9) $$W_{\left(u_{i},v_{i}\right)}\;=\;\left(\begin{array}{cccc} w_{i1} & 0 & \cdots & 0\\ 0 & w_{i2} & \cdots & 0\\ \vdots & \vdots & \ddots & \vdots \\ 0 & 0 & \cdots & w_{\mathrm{in}} \end{array}\right)$$

It is critical to select an appropriate weight matrix for estimating the parameters of GWR. The spatial weights can be estimated by a spatial kernel function, also called a distance-decay function. According to whether the bandwidth is varied, the 2 basic types of spatial kernels are fixed and adaptive kernels, which use fixed bandwidth and a fixed number of nearest neighbors within an adaptive bandwidth, respectively [33]. The commonly used spatial kernel functions include exponential kernel function, Gaussian kernel function, and bi-square kernel function [21]. In this study, the bi-square function was selected because it had the best (smallest) AICc for fitting the GWR model to the data. The adaptive kernel of bi-square function is defined as follows [34]:

(10) $$w_{ij}=\{\begin{array}{cc} {\left[1-{\left(\frac{d_{ij}}{h_{i}}\right)}^{2}\right]}^{2}, & if\;d_{ij}<h_{i}\\ 0\;, & otherwise \end{array}$$

where *d_ij_* is the distance between locations *i* and *j*; *h_i_* is the bandwidth used to estimate parameters at location *i*. The optimal bandwidth is usually selected based on a goodness-of-fit criterion such as cross-validation or Akaike Information Criterion (AIC) [25]. The corrected AIC (AICc) approach was applied for the optimal bandwidth selection in this study.

#### 2.2.3. GTWR and TWR Models

GTWR is an extension of GWR with temporal variations and incorporates both spatial and temporal heterogeneity in the data. The spatiotemporal nonstationary in the parameter estimates is captured by constructing the weight matrix based on spatiotemporal distances [24,25]. The GTWR model can be expressed as follows:

(11) $$Y_{i}=\beta_{0}\left(u_{i},v_{i},t_{i}\right)+\overset{p-1}{\underset{k=1}{\sum }}\beta_{k}\left(u_{i},v_{i},t_{i}\right)X_{ik}+\varepsilon_{i}\;\;\;\;\;\;\;i=1,\;2,\ldots ,n$$

The parameter $\beta_{k}\left(u_{i},v_{i},t_{i}\right)$ should be estimated for every predictor *k* and every space-time location *i*. The estimation of $\beta_{k}\left(u_{i},v_{i},t_{i}\right)$ is very similar to that in GWR (Equation (8)), and can be expressed as follows:

(12) $$\hat{\beta }\left(u_{i},v_{i},t_{i}\right)={\left(X^{T}W\left(u_{i},v_{i},t_{i}\right)X\right)}^{-1}X^{T}W\left(u_{i},v_{i},t_{i}\right)Y$$

Since GTWR considers both space and time, a spatial–temporal weight matrix is constructed based on a spatio–temporal distance. In this study, the Euclidean spatio–temporal distance was applied and determined as follows:

(13) $$d_{ij}^{ST}=\sqrt{\lambda \left[{\left(u_{i}-u_{j}\right)}^{2}+{\left(v_{i}-v_{j}\right)}^{2}\right]+\mu {\left(t_{i}-t_{j}\right)}^{2}}$$

where *λ* and *μ* are the scale factors in space and time metric system, respectively, which are used to balance the different effects that measure the spatial and temporal distance; *t_i_* and *t_j_* are the observed times at the locations *i* and *j*. In this study, an adaptive Gaussian distance–decay function was applied to construct a weight matrix. The weight matrix was still a diagonal matrix in GTWR and the diagonal elements are determined as follows [24]:

(14) $$w_{ij}=\exp\left\{-\frac{{\left(d_{ij}^{ST}\right)}^{2}}{h_{ST}^{2}}\right\}=\exp\left\{-\frac{{\left(d_{ij}^{S}\right)}^{2}}{h_{S}^{2}}\right\}\times \exp\left\{-\frac{{\left(d_{ij}^{T}\right)}^{2}}{h_{T}^{2}}\right\}$$

where $h_{ST}^{2}$ is a parameter of spatio–temporal bandwidth, and $h_{S}^{2}=h_{ST}^{2}/\lambda$ and $h_{T}^{2}=h_{ST}^{2}/\mu$ are spatial and temporal bandwidth, respectively. $d_{ij}^{S}=\sqrt{\left[{\left(u_{i}-u_{j}\right)}^{2}+{\left(v_{i}-v_{j}\right)}^{2}\right]}$ is the spatial distance, and $d_{ij}^{T}=\sqrt{{\left(t_{i}-t_{j}\right)}^{2}}$ is the temporal distance.

If no spatial variation exists in the observed data, the parameter *λ* would be zero (i.e., *λ* = 0) in Equation (13), that leads to the temporally weighted regression. TWR only considers the temporal variation based on the temporal distance. On the contrary, the parameter *μ* would be zero (i.e., *μ* = 0) in Equation (13) and GTWR would reduce to the traditional GWR without considering the temporal variation. Thus, both GWR and TWR are special cases of GTWR. All GWR-based local models (GWR, TWR, and GTWR) were performed using R package *Gwmodel* [35] under R version 3.5.1 [36].

#### 2.2.4. Model Assessment

The model goodness-of-fit was evaluated by the corrected Akaike’s information criterion (AICc) [21], adjusted coefficient of determination ($R_{a}^{2}$), root mean squared errors (RMSE), mean absolute error (MAE), and Z score of the model residuals. AICc is one of the most commonly used goodness-of-fit criteria for model comparisons and defined as Equation (15). The smaller the AICc is, the better the model performs.

(15) $$\mathrm{AICc}=2n\log_{e}\left(\hat{\sigma }\right)+n\log_{e}\left(2\pi \right)+n\left\{\frac{n+\mathrm{tr}\left(S\right)}{n-2-\mathrm{tr}\left(S\right)}\right\}$$

where *n* is the sample size, $\hat{\sigma }$ is the estimated standard deviation of the error term, and tr(*S*) denotes the trace of hat matrix *S* (i.e., $\hat{Y}=SY$). The hat matrix is a function of bandwidth and defined as follows in the GWR model:

(16) $$S=X{\left(X^{T}W\left(u_{i},v_{i}\right)X\right)}^{-1}X^{T}W\left(u_{i},v_{i}\right)$$

The coefficient of determination (*R*^2^) is another basic and widely used good-of-fit (Equation (17)). It represents the percentage of the total variation in the observed *Y* that is explained by the model. However, *R*^2^ tends to exaggerate the explained percentage since it never decreases by adding more predictor variables. The adjusted coefficient of determination ($R_{a}^{2}$) overcomes this drawback by dividing *RSS* and *SST* by their associated degrees of freedom (see Equation (18)). As multiple predictors were included, $R_{a}^{2}$ was adopted.

(17) $$R^{2}=1-\frac{RSS}{SST}$$

(18) $$R_{a}^{2}=1-\frac{RSS/\left(n-p\right)}{SST/\left(n-1\right)}=1-\frac{\left(n-1\right)\left(1-R^{2}\right)}{\left(n-p\right)}$$

where *RSS* is the residual sum of squares, and *SST* is the sum of squares of total variation of the response variable, *n* is the sample size, *p* is the number of coefficients (including all of predictor coefficients and intercept).

The *R*^2^ statistics of LMM is more complicated than the fixed-effects models and attracted many scientists’ attention [37,38]. In this study, the conditional *R*^2^ ($R_{c}^{2}$) was used, representing the variance explained by the entire model (including both fixed and random effects) [37], which can be expressed as follows:

(19) $$R_{c}^{2}=\frac{\sigma_{f}^{2}+\sigma_{r}^{2}}{\sigma_{f}^{2}+\sigma_{r}^{2}+\sigma_{\varepsilon }^{2}}$$

where $\sigma_{f}^{2}$, $\sigma_{r}^{2}$, and $\sigma_{\varepsilon }^{2}$ represent the variance of the fixed-effects, random-effects, and model residuals, respectively.

In addition to AICc and $R_{a}^{2}$, the root mean squared errors (RMSE), mean absolute error (MAE), and Z score of the model residuals are calculated to evaluate model performance by Equations (20−22), respectively:

(20) $$RMSE=\sqrt{\frac{\sum_{i=1}^{n}{\left(y_{i}-{\hat{y}}_{i}\right)}^{2}}{n}}$$

(21) $$MAE=\frac{\sum_{i=1}^{n}|y_{i}-{\hat{y}}_{i}|}{n}$$

(22) $$Z=\frac{y_{i}-{\hat{y}}_{i}}{std\left({\hat{y}}_{i}\right)}$$

where $y_{i}$ and ${\hat{y}}_{i}$ are the observed and predicted values of the response variable, and *std* denotes standard deviation.

The Moran’s *I* is commonly used to investigate the spatial dependencies in the model residuals from each regression model [23]. Moran’s I was positive when the model residuals tended to be similar, negative when they tended to be dissimilar, and approximately 0 when they arranged randomly and independently over space [39] and can be expressed as follows:

(23) $$I=\frac{n}{W}\cdot \frac{\sum_{i}\sum_{j}w_{ij}\left(e_{i}-\bar{e}\right)\left(e_{j}-\bar{e}\right)}{\sum_{i}{\left(e_{i}-\bar{e}\right)}^{2}}$$

where *w_ij_* is the diagonal elements of the spatial weight matrix, *W* is the sum of all *w_ij_*, *e_i_* denotes the residual at location *i* and $\bar{e}$ denotes the mean of residuals. The expected value of Moran’s I under the null hypothesis of no spatial autocorrelation (i.e., randomization) is $E\left(I\right)=\frac{-1}{n-1}$. In this study, the Moran’s I of the model residuals were calculated and plotted against the number of nearest neighbors (i.e., bandwidth) to present the relationship between spatial autocorrelation of the residuals and bandwidth. The *p*-value (two-tailed) calculated for the null hypothesis test of randomization was also plotted against the number of nearest neighbors.

## 3. Results

### 3.1. OLS and LMM

The OLS regression was used to model the relationships between PM_2.5_ and 5 criteria air pollutants as the benchmark for model comparisons. OLS was global and fixed-effects in nature and represented the average relationship between the response variable and predictors. Table 2 lists the parameter estimates of the OLS model (Equation (1)). All model coefficients were statistically significant and positive for predicting PM_2.5_, except O_3_. The OLS model indicated that PM_2.5_ increased as the 4 air pollutants (SO_2_, NO_2_, PM_10_, and CO) increased, while PM_2.5_ increased as O_3_ decreased. This was evident by the negative correlation between PM_2.5_ and O_3_ (Figure 2), which was consistent with a previous study [40]. Table 2 also indicated that PM_10_ was the most influential factor on PM_2.5_ because it had the largest standardized estimate (0.716). PM_2.5_ would increase 0.52 μg/m^3^ when PM_10_ increased 1 μg/m^3^ while keeping other predictors as constants. The OLS model fitted the data well according to the $R_{a}^{2}$, i.e., 85% of the total variation of PM_2.5_ can be explained by the OLS model.

The linear mixed model was applied for the 5 criteria air pollutants (SO_2_, NO_2_, PM_10_, CO, and O_3_) as the fixed-effects and the 13 cities (or region) as the random-effects. The G matrix (Equation (4)) of the random-effects was estimated by the covariance structure of variance components (VC), while the R matrix (Equation (4)) of the model residuals was estimated by the covariance structure of first-order autoregressive (AR(1)). The covariance structures of VC and AR(1) can be expressed by Equations (24) and (25),

(24) $$\mathrm{Var}\left(\gamma \right)=G=\left[\begin{array}{cccc} \sigma_{1}^{2} & 0 & \cdots & 0\\ 0 & \sigma_{2}^{2} & \cdots & 0\\ \vdots & \vdots & \ddots & \vdots \\ 0 & 0 & \cdots & \sigma_{6}^{2} \end{array}\right]$$

(25) $$\mathrm{Var}\left(\varepsilon \right)=R=\sigma^{2}\left[\begin{array}{cccccc} 1 & r & r^{2} & r^{3} & r^{4} & r^{5}\\ r & 1 & r & r^{2} & r^{3} & r^{4}\\ r^{2} & r & 1 & r & r^{2} & r^{3}\\ r^{3} & r^{2} & r & 1 & r & r^{2}\\ r^{4} & r^{3} & r^{2} & r & 1 & r\\ r^{5} & r^{4} & r^{3} & r^{2} & r & 1 \end{array}\right]$$

where $\sigma_{1}^{2}$ ~ $\sigma_{6}^{2}$ represent the variance of β_0_ (intercept), β_1_ (SO_2_), β_2_ (NO_2_), β_3_ (PM_10_), β_4_ (CO), and β_5_ (O_3_), respectively; $\sigma^{2}$ represents the variance of the model residuals, and *r* represents the first-order temporal autocorrelation. The parameter estimates of the G and R matrices were listed in Table 3. It seems that the temporal autocorrelations between the weekly observations were relatively high (*r* = 0.552) and significant.

Table 4 listed the parameter estimates of the fixed-effect (overall) of the LMM model. It indicated that all the coefficient estimates followed the same signs of the OLS estimates. The standard errors of the LMM coefficient estimates were much larger than those of the OLS estimates. This was because LMM treated the air pollutants as the fixed-effects but adjusted each coefficient estimate for each of the 13 cities (the random-effects), while OLS fitted an average model using the air pollutants, thus that underestimated the standard errors of the model coefficients. The LMM model explained 89.8% of the total variation of PM_2.5_ in the data. The AICc (18,756.7) of the LMM model was much smaller than that (20,109.26) of the OLS model, indicating a much better model fitting by LMM over OLS.

**Figure 4:**
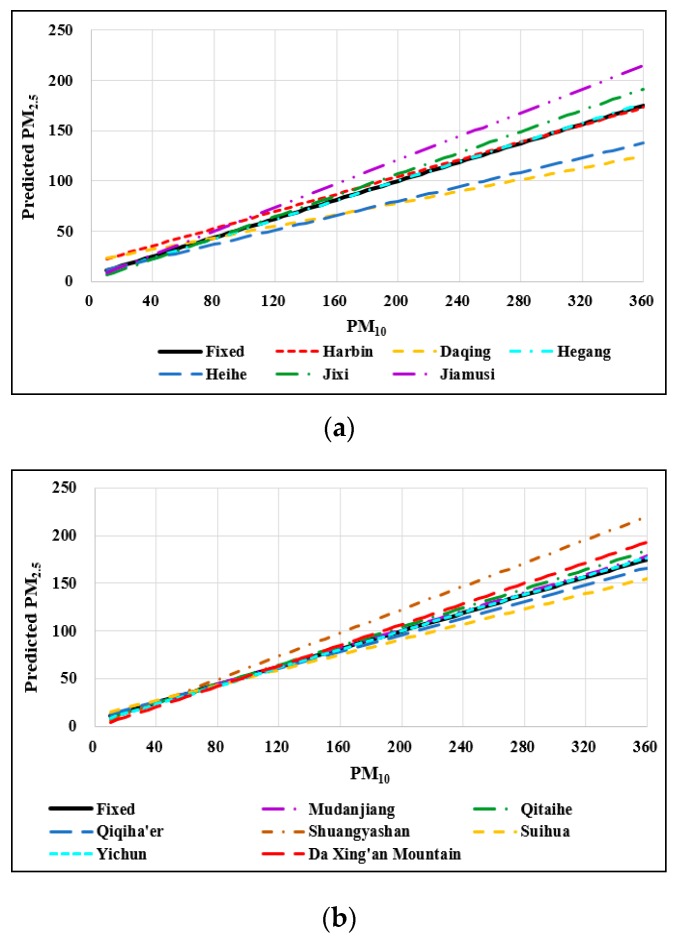
The relationship between particular matter (PM)_10_ and PM_2.5_ predicted by (**a**) fixed-effect (overall) vs. models of the 6 cities (**b**) fixed-effect (overall) vs. models of the other seven cities (region).

The 13 cities (or region) were treated as the random-effects in the LMM model thus that the model coefficients can be derived for each of the 13 cities (or region), which were listed in the Supplementary Materials (Table S1) due to the page limit. Figure 4 represents the relationships between predicted PM_2.5_ and the most significant predictor (PM_10_) as an example (while keeping other predictors as constants of means). The solid black line was the overall or fixed-effects model, while the colored dotted lines were the 13 models for the 13 cities (region). All the regression lines had slightly different slopes compared with the slope of the overall model. Among the 13 cities (region), the slope coefficient β_3_ (PM_10_) of Jiamusi (Figure 4a) and Shuangyashan (Figure 4b) were significantly greater (i.e., steeper slope) than that of the overall model, indicating that increasing the PM_10_ level in Jiamusi and Shuangyashan would cause a much higher PM_2.5_ than the other cities, especially for the higher concentrations of PM_10_. In contrast, Daqing (Figure 4a), Heihe (Figure 4a), and Suihua (Figure 4b) had significantly smaller β_3_ (i.e., shallower slope) than that of the overall model, indicating that increasing the PM_10_ level in Daqing, Heihe, and Suihua would cause a lower increase in PM_2.5_ than the other cities.

### 3.2. Local Models (GWR, TWR, and GTWR)

The local models, including GWR, TWR, and GTWR, were used to explore spatial and/or temporal heterogeneity in PM_2.5_ and 5 criteria air pollutants across the study area. The adaptive bi-square kernel function was used as the spatial weighting kernel function in this study. The appropriate bandwidth (number of neighbors) was selected based on the smallest AICc. The parameters and model fitting information of GWR, TWR, and GTWR are summarized in Table 5. The signs and magnitudes of the all median coefficients of the three GWR-based models (GWR, TWR, and GTWR) were compatible with those of the OLS model. However, the TWR’s model coefficients were more similar to those of GTWR, while GWR’s model coefficients were slightly different from those of TWR and GTWR.

### 3.3. Model Assessment

Table 6 showed the model goodness-of-fit (i.e., $R_{a}^{2}$ and AICc), prediction accuracy (i.e., RMSE and MAE), and prediction uncertainty (i.e., the mean and standard deviation (Std) of the Z score of model residuals). The three GWR-based models fitted the data better than the OLS model ($R_{a}^{2}$ = 0.846), with the order of TWR ($R_{a}^{2}$ = 0.968), GTWR ($R_{a}^{2}$ = 0.968), and GWR ($R_{a}^{2}$ = 0.884). Both GTWR and TWR also performed better than the LMM model ($R_{c}^{2}$ = 0.898), while GWR was slightly worse than LMM (Table 4). In the terms of AICc, TWR (17,944.31) fitted the data best, followed by GTWR (18,016.58), LMM (18,756.70), GWR (19,403.51), and OLS (20,109.26). The results indicated that the temporal autocorrelations in the 210-week time series data played a more important role than the spatial heterogeneity across the 13 cities (region). It was a bit surprising that LMM performed better than GWR in terms of goodness-of-fit. However, the prediction accuracy measures of GWR were slightly better than that of LMM in terms of RMSE (GWR 8.207 vs. LMM 8.482) and MAE (GWR 5.621 v.s. LMM 5.794). It was clear that TWR and GTWR were significantly superior to OLS, LMM and GWR according to all comparison criteria, i.e., they had higher $R_{a}^{2}$, smaller AICc, smaller RMSE, and MAE. TWR and GTWR reduced RMSE and MAE of the model residuals by 67% from the OLS model, while GWR only reduced RMSE and MAE by 15%.

**Figure 5:**
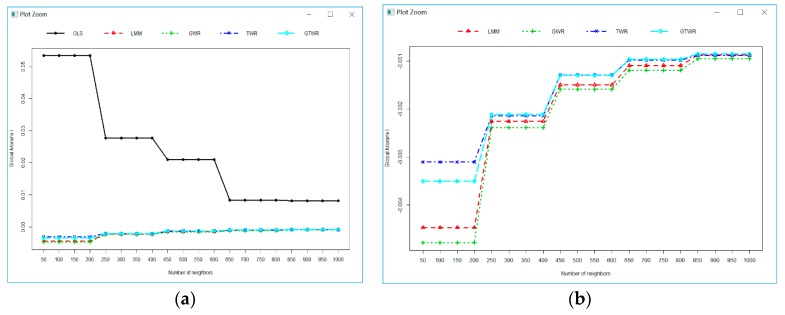
Moran’s I of the model residuals calculated using the different number of neighbors (**a**) OLS, LMM, GWR, TWR, and GTWR, and (**b**) only LMM, GWR, TWR, and GTWR.

To investigate the spatial dependencies in the model residuals from each model, the Moran’s I of the model residuals of OLS, GWR, TWR, and GTWR were calculated and compared. Figure 5 presents the Moran’s I calculated using different bandwidths (i.e., number of neighbors) for all 5 models (Figure 5a). The positive Moran’s I indicated that the model residuals were clustering in similar model residuals (either positive or negative), which may cause a serious violation of the independence assumption of the model residuals and lead to insufficient estimation of the model coefficients [39]. Therefore, the OLS model either under-predicted or over-predicted PM_2.5_ across the study area. The Moran’s I of the model residuals for all 5 models approached zero (i.e., randomness) with the increase of bandwidth (number of neighbors) and tended to be stable when the number of neighbors reached about 650.

However, the Moran’s I of the LMM and GWR-based models were much smaller than those of OLS and approached zero in the opposite direction (negative) to that of OLS (positive), which indicated that the residuals of LMM and GWR-based models were dissimilar to OLS when the bandwidth was small (Figure 5a). Figure 5b focused on the Moran’s I of LMM and GWR-based models only to see the differences between the 4 models. The LMM and GWR-based models generated little under-predictions or over-predictions for the patches of local areas and produced a significant reduction of spatial autocorrelation in the model residuals to various degrees by explicitly applying the local information among neighboring locations and/or time. The differences of Moran’s coefficients among LMM, GWR, TWR, and GTWR were more obvious with small bandwidths than large bandwidths. The LMM and GWR-based models had almost the same and little spatial autocorrelation when the number of neighbors was greater than 850. TWR performed over GTWR, and then both over LMM and GWR when the number of neighbors was less than 250. The performances of TWR and GTWR were almost the same when the bandwidth was larger than 250. It was consistent with the results of Table 6 that used the optimized bandwidth.

**Figure 6:**
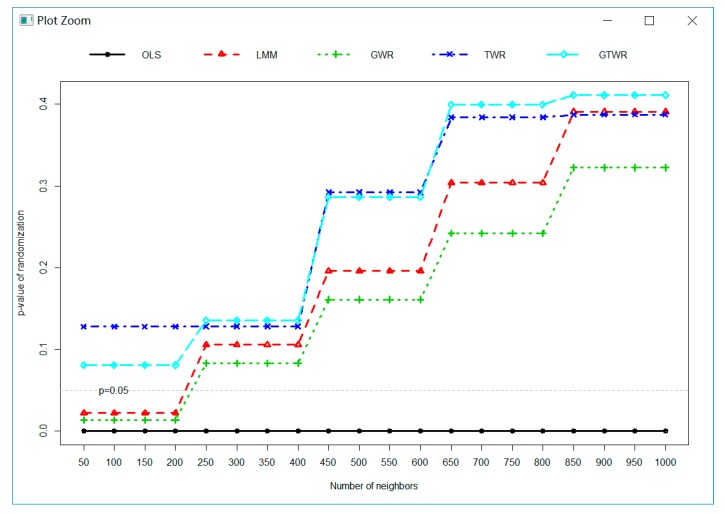
The *p*-values (two-tailed) for the randomization null hypotheses test of the model residuals of OLS, LMM, GWR, TWR, and GTWR calculated using a different number of neighbors.

Figure 6 presents the *p*-value (two-tailed) for the randomization null hypotheses test of the model residuals of OLS, LMM, GWR, TWR, and GTWR calculated using different bandwidths (i.e., number of neighbors). It indicated that the residuals of OLS were not random at any bandwidths since the *p*-value was much less than 0.05 (reject randomization null hypotheses) across all bandwidths. The residuals of LMM and all GWR-based models presented randomized character across all bandwidth except the residuals of LMM and GWR when the number of neighbors was smaller than 250. The magnitude of randomness gradually increased with the increase of the number of neighbors and followed by order of GWR, LMM, TWR, and GTWR. The TWR and GTWR had a similar trend. In general, the *p*-value of the randomization null hypotheses test followed the same trend of Moran’s coefficients since they all reacted to the spatial autocorrelation of the model residuals.

## 4. Discussion

Heilongjiang Province is the northernmost province in China, with the characters of high latitude, low temperature, four distinct seasons, and long and chilly winter. The cold winter leads to a very long heating season (from late October to March in the next year) in Heilongjiang. The industrial production, urban heating, emissions from unorganized pollution sources (such as straw burning) around cities, and motor vehicle exhaust emission mainly contributed to the air pollution of Heilongjiang [19]. Thus, effective measures should be implemented, such as the prohibition of open biomass burning, improvement of coal energy efficiency, and full use of clean fuels (nuclear, wind, and solar energy) for municipal heating [41].

**Figure 7:**
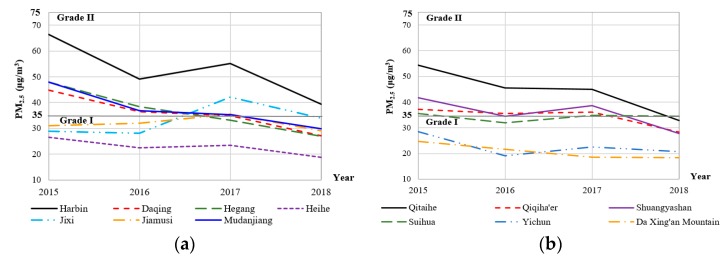
The annual concentration of PM_2.5_ of the 13 cities/regions of Heilongjiang Province from 2015 Table 2018. For visualization, (**a**) presents 7 cities, and (**b**) presents the other 6 cities. The grade I (35 µg/m^3^) and II (75 µg/m^3^) limits are shown. Note: For PM_2.5_ (μg/m^3^), grade I: ≤35, II: 35–75, III: 75–115, IV: 115–150, V: 150–250, VI: >250 (MEP, 2012).

In recent years, the Heilongjiang government has attached great importance to the fight of “blue sky defense” and made obvious progress in the prevention and control of air pollution. Fortunately, the annual concentration of PM_2.5_ from 2015 to 2018 presented a decreasing trend in most cities, except Jixi (Figure 7). The air pollutants are divided into 6 grades (I: Excellent; II: Good; III: Light pollution; IV: Moderate pollution; V: Heavy pollution; VI: Serious pollution) in terms of the Technical Regulation on Ambient Air Quality Index of China currently being tried [42,43]. Only Harbin (1 out of 13) exceeded the national annual PM_2.5_ standards (≤35 μg/m^3^) in all 4 years, but with a declining tendency within grade II. On the contrary, Heihe, Yichun, and Da Xing’an Mountain region were in excellent condition of air in all 4 years, which was consistent with the stable seasonal trend of the 6 air pollutants in Figure 3. The annual PM_2.5_ concentration of the other cities oscillated around the grade I limit (35 μg/m^3^). Some cities exceeded the grade I limit in 2015 and 2016 and then fell within the limit in the following 2 years (like Daqing, Hegang, and Mudanjiang), which indicated progress in the prevention and control of air pollution. However, Jixi, one of the most vital coal origins of the Heilongjiang Province, faced a more serious air pollution problem in 2017 than other years due to the poor air quality in heating seasons resulting from long and icy winters. This situation was alleviated in 2018. The provincial city Harbin had an even bigger air pollution problem than other cities because of the massive urban heating of around 9.5 million population, industry, and increased motor vehicle exhaust emissions every year. The phenomenon of spatial and temporal non-stationarity of PM_2.5_ shown in Figure 7 is consistent with that in Figure 3.

Recently, researchers have investigated the relationship between PM_2.5_ and various factors, such as aerosol optical depth (AOD) and normalized difference vegetation index (NDVI) derived from satellite imagery, meteorological factors (like temperature, wind speed, relatively humidity), transportation emission factors, density of industrial plants, land-use, gross domestic product (GDP), and digital elevation model (DEM) and so on [44,45,46,47,48,49,50]. These studies have led to an increasingly comprehensive understanding of PM_2.5_ and impact factors. Although PM_2.5_ correlates with many factors, it has the most direct correlation with the criteria air pollutants, especially PM_10_. It is meaningful to evaluate the spatial–temporal relationships between PM_2.5_ and criteria air pollutants since it can provide useful information on the PM_2.5_ concentration without direct PM_2.5_ monitoring, especially before 2015, when the PM_2.5_ monitoring network was sparse in Heilongjiang.

Meanwhile, PM_2.5_ modeling techniques vary according to the different predictors applied and objectives. In general, there are four major categories for modeling PM_2.5_: (1) Time series analysis and related statistical analysis (e.g., [16,51,52,53,54,55]); (2) GTWR and its derivative models (e.g., [30,56,57,58]); (3) machine learning method using plenty of predictors (e.g., [48,49]); (4) comprehensive approach by integrating several above methods (e.g., [46]). Each method has pros and cons. It is vital to choose an appropriate method instead of a complex approach to solve the problems according to a specific dataset. And, it is meaningful to compare different methods using the same dataset and accuracy indices.

In this study, the spatial and/or temporal heterogeneity of PM_2.5_ concentrations of Heilongjiang Province were investigated using LMM, GWR, TWR, and GTWR models based on the criteria air pollutants from 2015 to 2018. The major problem of global models applied to environmental processes is that they assume the processes to be constant across space or time. The spatial or temporal effects (spatial/temporal autocorrelation and heterogeneity) may violate the assumptions of independent observation and/or invariant variance, which biases the estimates of standard errors and results in imprecise coefficient estimates [59,60]. Although LMM is a global method, it could deal with spatial and temporal dependence and heterogeneity using appropriate variances of random-effects and random errors (i.e., G and R matrices), thus obtaining a better model performance than OLS (5% improvement of adjusted *R*^2^). However, the local model GWR did not perform better than LMM because it only handled spatial heterogeneity by “borrowing” the data from surrounding locations and ignored temporal information [25]. TWR and GTWR showed advantages over OLS by incorporating temporal heterogeneity (12% improvement of adjusted *R*^2^). The obvious seasonal variation (Figure 3) and decreasing tendency (Figure 7) lead to the high efficiency of incorporating temporal information in local models. However, the GTWR was not significantly superior to TWR in terms of the adjusted R^2^, AICc, RMSE, and MAE of the model residuals, which might result from the inadequate geographical locations (only 13 cities or regions). It was also the reason why the improvement of the GWR (3.8% of adjusted *R*^2^) was so limited. It is more efficient to incorporate temporal information than spatial information in this case. The criteria air quality data from all 56 air monitoring stations across the entire Heilongjiang Province could be applied to model the spatial and temporal heterogeneity of PM_2.5_. However, it is a challenge to process a massive dataset with both spatial and temporal information due to the limited calculation capability of the computer. Sometimes, it is a prerequisite to balance between the calculation efficiency and data richness. We sacrificed some spatial information by using the citywide average concentrations since they were the averages of concentrations at all monitoring sites in each city, and also the daily concentrations of air pollutants reported to the public by the government [61]. We also sacrificed some temporal information by aggregating daily data into weekly data for balancing those two. Nevertheless, the TWR model based on 210 weekly data was sufficient to describe the relationship between PM_2.5_ and the other 5 standard air pollutants in this study. Thus, it is scientific to apply the same policy for prevention and control of air pollution throughout the entire Heilongjiang Province with special attention paid to temporal changes.

Heilongjiang Province has begun to systematically and repeatedly measure the ambient air quality data (6 air pollutants) since 2015. How to reduce or eliminate the harm of PM_2.5_ to public health has become one of the most challenging problems for air pollution prevention in Heilongjiang Province. PM_2.5_ is inextricably related to other standard pollutants. Although some researchers have been investigated the spatial–temporal heterogeneity in the PM_10_–PM_2.5_ relationship using GWR-based models (e.g., [62]), the relationship between PM_2.5_ and criteria air pollutants (e.g., PM_10_, SO_2_, NO_2_, CO, O_3_) is still unclear and the spatial–temporal heterogeneity in the relationship is still to be studied.

## 5. Conclusions

In this study, we investigated the relationships between PM_2.5_ and 5 criteria air pollutants (i.e., PM_10_, SO_2_, NO_2_, CO, O_3_) for 13 cities (or region) of Heilongjiang Province during 2015~2018 using global and graphically and temporally weighted regression (i.e., OLS, LMM, GWR, TWR, and GTWR). The daily data were integrated into weekly data due to excessive computation. The model performance and spatial autocorrelation of model residuals were compared. The results showed that all parameter estimates tended to be positive for predicting PM_2.5_, except O_3_. The LMM and all GWR-based models (i.e., GWR, TWR, and GTWR) showed great advantages over OLS in terms of model fitting, such as producing higher R^2^ and more desirable model residuals, especially TWR and GTWR that incorporated temporal variation. The GWR, LMM, and TWR and GTWR improved the explanation of variance in PM_2.5_ by 3%, 5%, and 12%, respectively, from the R^2^ (0.85) of OLS. TWR yielded slightly better model performance, prediction accuracy and uncertainty accuracy than GTWR (smaller AICc, RMSE, MAE and standard deviation of Z score of model residuals), and also reduced RMSE and MAE of model residuals by 67% comparing to OLS model; while GWR only reduced RMSE and MAE by 14%~15% comparing to OLS. The traditional OLS and GWR were inadequate for describing the nonstationary of PM_2.5_. The LMM slightly performed better than GWR since it considered different locations as a random effect and meanwhile handled the repeated measurements using the *R* matrix, which provided an alternative solution besides the GWR-based models. Although GWR that incorporates the spatial non-stationarity of PM_2.5_ improved the model performance of OLS, it is still far from TWR that incorporates temporal nonstationary of PM_2.5_. GTWR did not bring any improvements to TWR by adding spatial information because of the limited number of locations. The temporal heterogeneity is more obvious than spatial heterogeneity in this case. Thus, the incorporation of temporal information is inevitable and adequate for modeling the relationship between PM_2.5_ and the other air pollutants in this study. This work provides evidence of spatial–temporal heterogeneity in the relationship between PM_2.5_ and the other standard air pollutants, and also provides possible solutions for modeling PM_2.5_ with the other air pollutants for Heilongjiang province. In addition, the localized model coefficients and predictions of the GWR models can provide spatio-temporal “hot spots” of PM_2.5_ pollution, which should be useful for assisting the governmental agencies to pin-point the seriousness of air pollution or local emission in order to make better management decisions.

## Figures and Tables

**Table 1:** Descriptive statistics of the variables used in this study (*n* = 2730).

Variables	Min.	Q_1_	Mean	Median	Q_3_	Max.	Std
SO_2_ (μg/m^3^)	2.00	7.57	16.48	12.00	21.29	178.00	14.17
NO_2_ (μg/m^3^)	2.86	15.43	22.95	20.29	28.29	104.43	11.12
PM_10_ (μg/m^3^)	10.71	36.00	58.67	50.21	71.57	363.86	33.69
CO (mg/m^3^)	0.10	0.50	0.72	0.64	0.87	3.03	0.34
O_3_ (μg/m^3^)	15.57	54.00	72.16	68.64	87.00	172.29	23.81
PM_2.5_ (μg/m^3^)	3.57	17.86	34.09	26.43	42.71	235.57	24.50

**Table 2:** Parameter estimates of the ordinary least squares regression (OLS) model.

Parameter	Estimate	Std. Error	t Test	*p*-Value	Standardized Estimate
Intercept	−4.398	0.850	−5.173	0.000	0.000
SO_2_ (μg/m^3^)	0.081	0.017	4.679	0.000	0.047
NO_2_ (μg/m^3^)	0.380	0.025	15.368	<2 × 10^−16^	0.172
PM_10_ (μg/m^3^)	0.520	0.007	69.733	<2 × 10^−16^	0.716
CO (mg/m^3^)	5.640	0.715	7.884	0.000	0.079
O_3_ (μg/m^3^)	−0.086	0.008	−10.221	<2 × 10^−16^	−0.083
**Model fitting information**					
$R_{a}^{2}$			0.846		
AICc			20,109.26		

**Table 3:** Estimates of the variance of linear mixed models (LMM).

Parameter	$\sigma_{1}^{2}$	$\sigma_{2}^{2}$	$\sigma_{3}^{2}$	$\sigma_{4}^{2}$	$\sigma_{5}^{2}$	$\sigma_{6}^{2}$	*r*	$\sigma^{2}$
Estimate	78.092	0.022	0.082	0.009	157.010	0.001	0.552	75.110

**Table 4:** Parameter estimates of overall (fixed-effect) of LMM.

Parameter	Estimate	Std. Error	t Test	*p*-Value
Intercept	−9.81	2.64	−3.71	0.00
SO_2_ (μg/m^3^)	0.07	0.05	1.27	0.23
NO_2_ (μg/m^3^)	0.42	0.09	4.78	0.00
PM_10_ (μg/m^3^)	0.47	0.03	17.27	<0.0001
CO (mg/m^3^)	11.86	3.62	3.28	0.01
O_3_ (μg/m^3^)	−0.05	0.01	−3.54	0.00
**Model fitting information**				
$R_{c}^{2}$		0.898		
AICc		18,756.70		

Note: $R_{c}^{2}$ is the conditional *R*^2^ of LMM.

**Table 5:** Parameter estimates of geographically weighted regression (GWR), temporally weighted regression (TWR), and geographically and temporally weighted regression (GTWR).

Models	Parameter	Min	Q_1_	Median	Q_3_	Max	Model fitting Information
GWR (Num of Neighbors = 262; Adaptive)	Intercept	−26.901	−10.534	−7.609	−3.836	5.662	$R_{a}^{2}$: 0.884 AICc: 19,403.51
	SO_2_	−0.270	−0.021	0.046	0.259	0.516	
	NO_2_	−0.282	0.084	0.400	0.676	1.061	
	PM_10_	0.325	0.437	0.536	0.586	0.628	
	CO	−4.847	1.834	3.867	11.796	34.484	
	O_3_	−0.171	−0.102	−0.075	−0.026	0.052	
TWR (Num of Neighbors = 20; Adaptive)	Intercept	−99.573	−14.089	−5.141	2.918	91.439	$R_{a}^{2}$: 0.968 AICc: 17,944.31
	SO_2_	−2.985	−0.199	0.099	0.535	4.196	
	NO_2_	−2.667	−0.234	0.136	0.551	2.637	
	PM_10_	−0.259	0.283	0.471	0.704	1.140	
	CO	−41.876	−3.172	6.125	19.781	137.483	
	O_3_	−1.250	−0.116	−0.017	0.065	0.918	
GTWR (Num of Neighbors = 20; Adaptive)	Intercept	−99.573	−14.412	−5.339	2.790	91.439	$R_{a}^{2}$: 0.968 AICc: 18016.58
	SO_2_	−2.985	−0.197	0.112	0.550	4.196	
	NO_2_	−2.667	−0.232	0.150	0.582	2.637	
	PM_10_	−0.259	0.279	0.456	0.700	1.140	
	CO	−53.703	−3.128	6.111	19.953	137.483	
	O_3_	−1.250	−0.118	−0.017	0.065	0.918	

Note: The unit of SO_2_, NO_2_, and PM_10_ and O_3_ is μg/m^3^, the unit of CO is mg/m^3^.

**Table 6:** Comparison of OLS, LMM, and GWR-based models (GWR, TWR, and GTWR).

Num of Neighbor	Model Type	AICc	$R_{a}^{2}$	RMSE of Residuals	MAE of Residuals	Mean of Z Score	Std of Z Score
——	OLS	20,109.25	0.846	9.598	6.587	0	0.426
——	LMM	18,757.5	0.898 ^§^	8.482	5.794	−0.001	0.375
262	GWR	19,403.49	0.884	8.207	5.621	0	0.356
20	TWR	17944.39	0.968	3.13	2.175	0	0.129
20	GTWR	18016.58	0.968	3.16	2.187	0	0.131

Note: “^§^” represents the conditional *R*^2^ ($R_{c}^{2}$).

## Supplementary Materials

The following are available online at https://www.mdpi.com/1660-4601/16/24/5107/s1, Table S1: Parameter estimates of random effect and final estimates of LMM.
