# Supplementary material for: Global and Geographically and Temporally Weighted Regression Models for Modeling PM2.5 in Heilongjiang, China from 2015 to 2018
Source: Int J Environ Res Public Health. 2019 Dec 14;16(24):5107. doi: 10.3390/ijerph16245107 (PMC6950195; doi:10.3390/ijerph16245107)
Supplement: Supplementary file 1 [file ijerph-16-05107-s001.zip › Table S1.docx]

Table S1. Parameter estimates of random effect and final estimates of LMM

|  |  | Random effect | | Final estimate |  | Random effect | | Final estimate |
| --- | --- | --- | --- | --- | --- | --- | --- | --- |
| City | Parameters | Estimate | p-value |  | City | Estimate | p-value |  |
| 1 | Intercept | -21.60 | <.0001 | -31.40 | 8 | -1.60 | 0.66 | -11.41 |
|  | SO2 | 0.01 | 0.87 | 0.08 |  | 0.14 | 0.19 | 0.21 |
|  | NO2 | 0.31 | 0.01 | 0.73 |  | -0.31 | 0.01 | 0.11 |
|  | PM10 | -0.04 | 0.20 | 0.43 |  | 0.03 | 0.37 | 0.50 |
|  | CO | 8.32 | 0.07 | 20.18 |  | 12.11 | 0.01 | 23.97 |
|  | O3 | -0.01 | 0.69 | -0.06 |  | -0.04 | 0.07 | -0.09 |
| 2 | Intercept | -11.51 | 0.00 | -21.32 | 9 | -3.85 | 0.29 | -13.65 |
|  | SO2 | 0.09 | 0.39 | 0.16 |  | 0.08 | 0.33 | 0.15 |
|  | NO2 | 0.01 | 0.92 | 0.43 |  | 0.38 | 0.02 | 0.80 |
|  | PM10 | -0.18 | <.0001 | 0.29 |  | -0.03 | 0.42 | 0.44 |
|  | CO | 31.85 | <.0001 | 43.71 |  | -5.19 | 0.32 | 6.68 |
|  | O3 | 0.04 | 0.11 | -0.01 |  | -0.02 | 0.54 | -0.06 |
| 3 | Intercept | 8.32 | 0.02 | -1.49 | 10 | 1.13 | 0.76 | -8.68 |
|  | SO2 | 0.00 | 0.97 | 0.07 |  | -0.06 | 0.58 | 0.01 |
|  | NO2 | -0.41 | 0.00 | 0.01 |  | 0.14 | 0.37 | 0.56 |
|  | PM10 | 0.01 | 0.81 | 0.48 |  | 0.14 | 0.00 | 0.61 |
|  | CO | -1.51 | 0.72 | 10.36 |  | -10.87 | 0.03 | 0.99 |
|  | O3 | -0.01 | 0.74 | -0.06 |  | -0.01 | 0.78 | -0.06 |
| 4 | Intercept | 5.00 | 0.17 | -4.81 | 11 | 0.39 | 0.91 | -9.42 |
|  | SO2 | 0.11 | 0.24 | 0.18 |  | -0.22 | 0.00 | -0.15 |
|  | NO2 | -0.14 | 0.40 | 0.28 |  | 0.35 | 0.00 | 0.77 |
|  | PM10 | -0.11 | 0.01 | 0.36 |  | -0.07 | 0.01 | 0.40 |
|  | CO | -2.08 | 0.69 | 9.78 |  | -0.84 | 0.85 | 11.03 |
|  | O3 | 0.03 | 0.17 | -0.01 |  | 0.03 | 0.23 | -0.02 |
| 5 | Intercept | 2.91 | 0.40 | -6.90 | 12 | 8.26 | 0.02 | -1.55 |
|  | SO2 | -0.19 | 0.03 | -0.12 |  | 0.02 | 0.90 | 0.08 |
|  | NO2 | 0.19 | 0.20 | 0.60 |  | -0.15 | 0.30 | 0.27 |
|  | PM10 | 0.06 | 0.10 | 0.53 |  | 0.01 | 0.90 | 0.48 |
|  | CO | -9.57 | 0.02 | 2.30 |  | -6.01 | 0.24 | 5.85 |
|  | O3 | -0.03 | 0.23 | -0.08 |  | 0.01 | 0.78 | -0.04 |
| 6 | Intercept | 4.21 | 0.24 | -5.60 | 13 | 5.64 | 0.14 | -4.17 |
|  | SO2 | 0.04 | 0.72 | 0.11 |  | -0.12 | 0.16 | -0.05 |
|  | NO2 | -0.10 | 0.45 | 0.32 |  | -0.01 | 0.95 | 0.41 |
|  | PM10 | 0.12 | 0.00 | 0.59 |  | 0.07 | 0.12 | 0.54 |
|  | CO | -8.05 | 0.07 | 3.82 |  | -12.36 | 0.00 | -0.49 |
|  | O3 | 0.00 | 0.85 | -0.04 |  | 0.01 | 0.76 | -0.04 |
| 7 | Intercept | 2.71 | 0.46 | -7.10 |  |  |  |  |
|  | SO2 | 0.08 | 0.42 | 0.15 |  |  |  |  |
|  | NO2 | -0.27 | 0.05 | 0.14 |  |  |  |  |
|  | PM10 | 0.01 | 0.85 | 0.48 |  |  |  |  |
|  | CO | 4.20 | 0.43 | 16.06 |  |  |  |  |
|  | O3 | 0.00 | 0.89 | -0.05 |  |  |  |  |

Note: City index: 1-Harbin; 2-Daqing; 3-Hegang; 4-Heihe; 5-Jixi; 6-Jiamusi; 7-Mudanjiang; 8-Qitaihe; 9-Qiqiha’er; 10-Shuangyashan; 11-Suihua; 12-Yichun; 13-Da Xing’an Mountain region.
